# Supplementary material for: Active site specificity profiling datasets of matrix metalloproteinases (MMPs) 1, 2, 3, 7, 8, 9, 12, 13 and 14
Source: Data Brief. 2016 Feb 22;7:299–310. doi: 10.1016/j.dib.2016.02.036 (PMC4777984; doi:10.1016/j.dib.2016.02.036)
Supplement: Supplementary file 10 — Supplementary material [file mmc10.zip › WebPICS_hMMP9_T_1%/P1.html]

 

PICS results


|  |  |
| --- | --- |
| **P1\_A**  28 in 138 sites   20.3 %    effects > 10 perc. pnts.  (vice-versa in brackets)  P3prime\_A: 13.2 (12.0) |  |
| **P1\_C**  3 in 138 sites   2.2 %    effects > 10 perc. pnts.  (vice-versa in brackets)  P1prime\_M: 29.0 (14.5) |  |
| **P1\_D**  9 in 138 sites   6.5 %    effects > 10 perc. pnts.  (vice-versa in brackets)  P3\_V: 23.9 (16.6)   P2\_V: 26.1 (23.5)   P2prime\_M: 16.4 (18.5) |  |
| **P1\_E**  10 in 138 sites   7.2 %    effects > 10 perc. pnts.  (vice-versa in brackets)  P2\_G: 12.0 (11.0)   P3prime\_S: 23.3 (10.2) |  |
| **P1\_G**  22 in 138 sites   15.9 %    effects > 10 perc. pnts.  (vice-versa in brackets)  P1prime\_I: -14.3 (-12.1) |  |
| **P1\_H**  4 in 138 sites   2.9 %    effects > 10 perc. pnts.  (vice-versa in brackets)  P2\_Y: 19.9 (11.4)   P2prime\_Q: 39.9 (11.4)   P3prime\_H: 20.7 (13.8) |  |
| **P1\_N**  10 in 138 sites   7.2 %    effects > 10 perc. pnts.  (vice-versa in brackets)  P3\_A: 20.6 (15.9)   P2\_Q: 14.2 (17.8)   P2\_Y: 14.9 (21.4)   P1prime\_M: 15.7 (26.1)   P2prime\_V: 28.4 (17.8) |  |
| **P1\_P**  12 in 138 sites   8.7 %    effects > 10 perc. pnts.  (vice-versa in brackets)  P2\_F: 12.4 (24.6)   P1prime\_Q: 10.2 (13.5)   P2prime\_T: 10.9 (16.3)   P3prime\_H: 12.4 (24.6) |  |
| **P1\_Q**  9 in 138 sites   6.5 %    effects > 10 perc. pnts.  (vice-versa in brackets)  P2\_G: 14.2 (11.7)   P2prime\_I: 22.4 (13.5) |  |
| **P1\_S**  14 in 138 sites   10.1 %    effects > 10 perc. pnts.  (vice-versa in brackets)  P3\_A: 12.0 (13.0)   P2\_S: 15.6 (12.1)   P2\_Y: 16.3 (32.8)   P2prime\_H: 14.2 (19.9) |  |
| **P1\_W**  3 in 138 sites   2.2 %    effects > 10 perc. pnts.  (vice-versa in brackets)  P2\_H: 29.0 (14.5)   P1prime\_W: 31.1 (31.1)   P2prime\_T: 27.5 (10.3)   P3prime\_H: 29.0 (14.5) |  |
